# The Tokyo GIM Conference: Clinical reasoning conference from real cases

**DOI:** 10.1002/jgf2.209

**Published:** 2018-09-24

**Authors:** Satoshi Watanuki, Ryuichi Sada, Masahiro Ishikane, Taro Shimizu, Satoshi Kutsuna

**Affiliations:** ^1^ Division of Emergency and General Medicine Tokyo Metropolitan Tama Medical Center Tokyo Japan; ^2^ Department of General Internal Medicine Kameda Medical Center Chiba Japan; ^3^ Disease Control and Prevention Center National Center for Global Health and Medicine Tokyo Japan; ^4^ Department of Diagnostic and Generalist Medicine Dokkyo Medical University Tochigi Japan

## Abstract

The view of Tokyo GIM Conference.

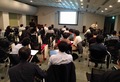

Clinical reasoning seminars using real cases are effective for improving clinical problem‐solving skills because real cases reflect the false leads, polymorphisms in actual clinical materials, and misleading test results encountered in daily practice.^1^ However, opportunities for undergraduate and postgraduate education in clinical reasoning using real cases are scarce in Japan. In order to improve this situation, we established the Tokyo General Internal Medicine (GIM) Conference in the Kanto Region in 2011. We would like to describe the management style and effectiveness of this seminar.

The Tokyo GIM Conference is held every second Friday night at Shinjuku in central Tokyo with the aim of providing learning opportunities in clinical reasoning. Case presentation is scheduled chiefly by the organizers and is conducted two or three times per month. The organizers serve as moderators and facilitators and are responsible for eliciting comments from the participants and explanations from the experts.

The Tokyo GIM Conference is a bona fide organization and receives no financial support from the pharmaceutical industry. All publicity for the Tokyo GIM Conference is conducted via a social network (Facebook), and short case series in Japanese focusing on addressing clinical challenges are published in a medical periodical by Igaku‐Shoin. The venues for the conference are provided free of charge by the home institution of the organizer or case presenter. Regarding the load for staff, the time and labor provided by the organizers contribute largely to making the conference possible.

To date, we have held this conference at over 20 venues throughout the Kanto Region. About 50 senior medical students and young physicians up to 10 years after graduation(See Figure 1) participate in each seminar. Case presentations are extremely educational because of diagnostic difficulties or diagnostic errors encountered in real cases. The main diagnostic errors consist of diagnostic delay, misdiagnosis, and wrong tentative diagnosis.

**Figure 1:**
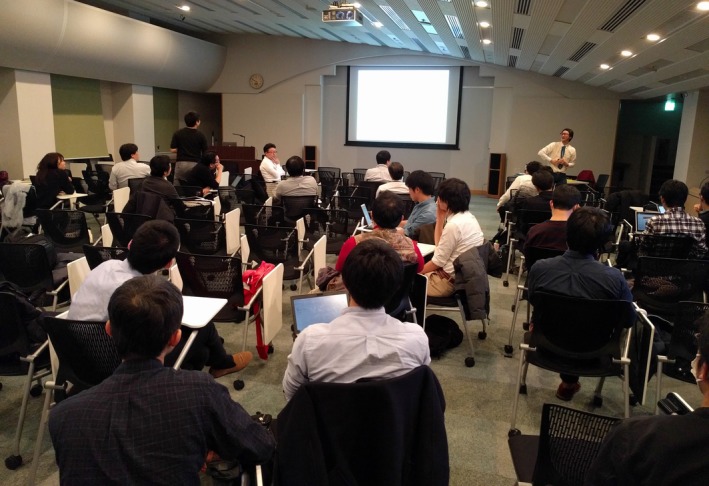
The view of The Tokyo GIM Conference

A group interview conducted by the organizers examines which factors are most important for continuing interest in the conference. The interview exemplifies the following merits. First, this seminar is unique because no other similar opportunity exists in the Kanto Region. Second, the quality of the content is very high because every case presentation has teaching points. Third, the scheduling and structure are based on those of the Kyoto GIM Conference. And finally, the seminar is held in places with convenient access.

In conclusion, our aim was to provide busy Japanese medical professionals with the convenient opportunity to participate in our educational seminar to improve their clinical problem‐solving skills. Our future plans include expanding our seminar throughout Japan via the Internet.

## CONFLICT OF INTEREST

The authors have stated explicitly that there are no conflicts of interest in connection with this article.
